# Manifestations of immune tolerance in the human female reproductive tract

**DOI:** 10.3389/fimmu.2013.00026

**Published:** 2013-02-13

**Authors:** Gary F. Clark, Danny J. Schust

**Affiliations:** Department of Obstetrics, Gynecology and Women's Health, Division of Reproductive Medicine and Fertility, University of MissouriColumbia, MO, USA

**Keywords:** cervix, semen, trophoblast, immune privilege, human, vagina

## Abstract

Like other mucosal surfaces (e.g., the gastrointestinal tract, the respiratory tract), the human female reproductive tract acts as an initial barrier to foreign antigens. In this role, the epithelial surface and subepithelial immune cells must balance protection against pathogenic insults against harmful inflammatory reactions and acceptance of particular foreign antigens. Two common examples of these acceptable foreign antigens are the fetal allograft and human semen/sperm. Both are purposely deposited into the female genital tract and appropriate immunologic response to these non-self antigens is essential to the survival of the species. In light of the weight of this task, it is not surprising that multiple, redundant and overlapping mechanisms are involved. For instance, cells at the immunologic interface between self (female reproductive tract epithelium) and non-self (placental trophoblast cells or human sperm) express glycosylation patterns that mimic those on many metastatic cancer cells and successful pathogens. The cytokine/chemokine milieu at this interface is altered through endocrine and immunologic mechanisms to favor tolerance of non-self. The “foreign” cells themselves also play an integral role in their own immunologic acceptance, since sperm and placental trophoblast cells are unusual and unique in their antigen presenting molecule expression patterns. Here, we will discuss these and other mechanisms that allow the human female reproductive tract to perform this delicate and indispensible balancing act.

## Introduction

There are specific locations in human tissues and organs where alloantigens and autoantigens are tolerated by the immune system. This tolerance can exist indefinitely or for defined periods of time like pregnancy. This uncoupling of the adaptive immune response confers a physiological state known as immune privilege (Streilein, 1995). Evidence suggesting the existence of immune privilege was first obtained by van Dooremal, who documented the extended survival of murine skin xenografts in the anterior chamber of the dog eye in 1873 (van Dooremaal, 1873). Other well-established immune privileged tissues and organs include the uteroplacental unit (Medawar, 1953; Beer and Billingham, 1971), brain (Muldoon et al., 2013), testes (Li et al., 2012), and the prostate (Neaves and Billingham, 1979; Leibovitz et al., 2004).

Peter Medawar initially recognized that the mammalian fetus is an allograft due to the contribution of its foreign paternal alloantigens (Medawar, 1953). Unrelated surrogate mothers readily accommodate a completely foreign fetus as well as their own offspring, confirming that maternal histocompatibility is unnecessary. These observations indicate that the female reproductive tract is immune privileged during pregnancy. However, a sexually active woman's immune components must respond robustly to pathogens at all times, yet remain selectively tolerant to male-associated antigens present in human seminal plasma and sperm. The goal of this review is to consider the current evidence describing how immune privilege is manifested in the female reproductive tract during pregnancy and semen exposure, both of which should otherwise provoke potent immune responses.

## Immune privilege during pregnancy

### The human zona pellucida (ZP)

The mammalian egg is surrounded by the zona pellucida (ZP), which acts as a specialized extracellular matrix for binding sperm. For fertilization to occur, sperm must first bind to this matrix, transit through this barrier and fuse with the egg cell to form a zygote (Clark, 2010). The zygote is an equal combination of both the maternal and foreign paternal genomes. It is unknown at which stage the human pre-embryo begins to express paternal major histocompatibility (MHC) molecules. However, in the mouse, MHC expression is readily detected at the eight cell stage (Ewoldsen et al., 1987). Cytotoxic T lymphocyte cells (CTL) sensitized to paternal MHC antigens are unable to kill mouse pre-embryos surrounded by an intact ZP, but pre-embryos denuded of this matrix are immediately destroyed (Ewoldsen et al., 1987).

**Figure 1:**
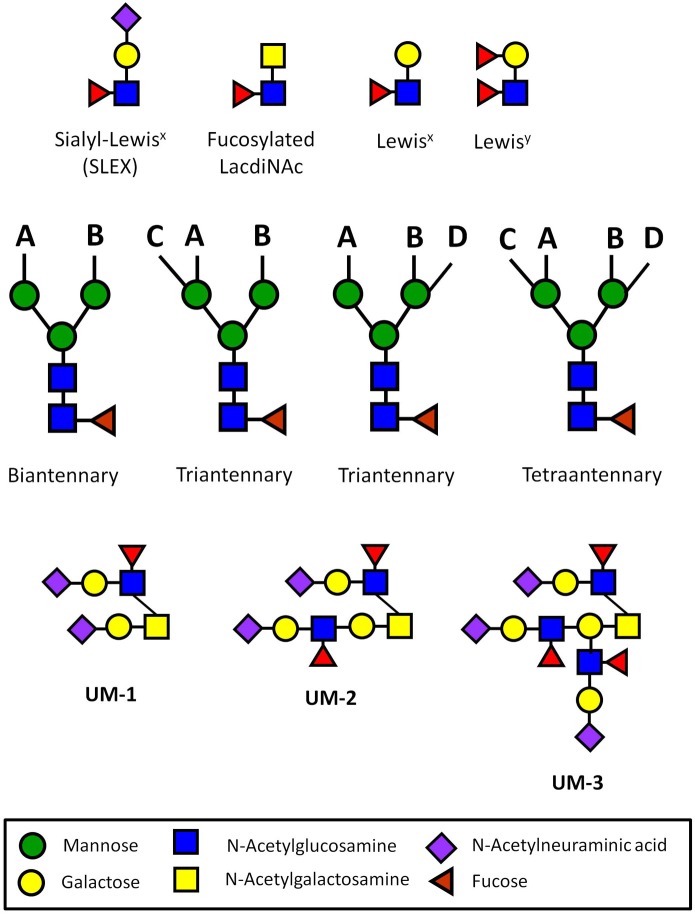
**Carbohydrate sequences involved in immune privilege in the human reproductive system.** N-glycans usually have two (biantennary), three (triantennary), or four (tetraantennary) antennae linked at up to four positions (designated A–D). On the human ZP, there are biantennary and triantennary N-glycans terminated on every antenna with the SLEX sequences on every antenna (Pang et al., 2011). Tetraantennary N-glycans bearing three SLEX antenna are also present. Human sperm and seminal plasma express bi-, tri-, and tetra-antennary N-glycans terminated exclusively with Lewis^x^ or exclusively with Lewis^y^ sequences on their antennae, though many carry a mixture of both of these sequences (Pang et al., 2007, 2009). Glycodelin-A bears the fucosylated lacdiNAc sequence on 60% of its total N-glycans (Dell et al., 1995). Uromodulin expresses one (UM-1), two (UM-2), or three SLEX sequences on a single O-glycan (Easton et al., 2000). These types of presentations have not been found in other normal cells or tissues outside of the human reproductive system.

The ZP has been suggested to simply act as a physical barrier against the rejection of the human pre-embryo (Ewoldsen et al., 1987). However, many types of immune cells transit through similar types of physical barriers during the rejection of a foreign organ transplant (Krensky et al., 1990). Human ZP glycoproteins express N-glycans terminated with multivalent sialyl-Lewis^x^ sequences (SLEX) that mediate sperm binding (Figure 1) (Pang et al., 2011). SLEX is also the universal ligand for the selectins, cell adhesion molecules that mediate both the binding of immune cells to inflamed vascular endothelium and lymphocyte homing (Foxall et al., 1992; Fukuda et al., 1999). SLEX is also a ligand for siglec-9, an immunoglobulin-like lectin that carries an immunoreceptor tyrosine-based inhibitory motif (ITIM) that generates an inhibitory signal in several immune cell populations (Angata and Varki, 2000; Avril et al., 2004). The possibility has been raised that the carbohydrate sequences expressed on the ZP act as functional groups to protect the early pre-embryo before blastocyst hatching (Clark et al., 1996, 1997). In short, the human egg itself could be an immune privileged cell type both before and after fertilization.

### Glycodelin-a (Gda) and CA125

When the histoincompatible human pre-embryo hatches out of the blastocyst, it faces the daunting task of invading the maternal endometrial lining where four major immune cell populations are present: uterine NK cells (uNK), macrophages, T cells, and dendritic cells (DC) (King et al., 1998; Tirado-Gonzalez et al., 2012). During the early stages of implantation, trophoblast cells secrete the chemokine MIP-1α that induces uNK cells and monocytes to migrate to the implantation site, resulting in the formation of a dense infiltrate of these cells in the decidua basalis (Drake et al., 2001). It is apparent that the implanting human embryo is challenged at an early stage by these immune cells. One major question becomes apparent: how does the encircled human embryo resist challenge by these immune cells at this early stage?

Glycodelin-A (GdA) (PP14) is a luteal phase endometrial glycoprotein that is secreted beginning 2 days after ovulation (Dalton et al., 1995). This 27 kDa glycoprotein manifests many different immune deviating effects when present at physiological concentrations *in vitro* (Table 1). If implantation succeeds, the synthesis of GdA is massively induced, becoming 4–16% of the total protein content expressed in early stage decidua (7–11 weeks of pregnancy) (Julkunen et al., 1985). GdA is also taken up by the placenta and concentrated in this organ. This glycoprotein is also present in physiologically relevant concentrations in amniotic fluid, reaching levels averaging 46 μg/ml between 12 and 20 weeks (Julkunen et al., 1985). However, the level of GdA decreases dramatically after 20 weeks, becoming a minor component of decidual proteins and the amniotic fluid at term.

**Table 1:** **Immunomodulatory activities of Glycodelin-A**.

**Effect**	**References**
Inhibits T cell proliferation by PHA and other activators	Pockley et al., 1988
Decreases production of IL-2 following T cell activation	Pockley and Bolton, 1989
Induces apoptosis of activated T cells	Mukhopadhyay et al., 2001
Binds CD45 on T cells via a potential lectin-like activity	Ish-Shalom et al., 2006
Inhibits lysis of K562 target cells by large granular lymphocytes	Okamoto et al., 1991
Diminishes IgM secretion and MHC class II expression in B cells	Yaniv et al., 2003
Blocks chemoattractant induced migration of monocytes	Mukhopadhyay et al., 2001
Inhibitor of E-selectin-mediated cell adhesion	Jeschke et al., 2003
Stimulates IL-6 secretion by monocytes/ macrophages via interaction with L-selectin and the extracellular signal regulated kinase pathway	Lee et al., 2012

Glycodelin has also been isolated from seminal plasma and has been designated GdS. Its protein backbone is identical to GdA, but GdS does not cause the diverse immunomodulatory effects associated with GdA. Instead, *in vitro* studies indicate that it blocks the capacitation of human sperm (Chiu et al., 2005). Biophysical analyses of GdA and GdS confirm major differences in their N-glycosylation patterns. GdA expresses very unusual fucosylated lacdiNAc and Sd^a^ sequences on the antennae of its N-glycans; these are completely lacking in GdS (Morris et al., 1996; Lee et al., 2009). The fucosylated lacdiNAc sequence is a carbohydrate ligand for both selectins and DC-SIGN, two immune lectins that have been implicated in leukocyte/lymphocyte binding and the modulation of the adaptive immune response, respectively (Grinnell et al., 1994; van Liempt et al., 2006). The carbohydrate sequences linked to GdA have been implicated as functional groups that enable this glycoprotein to mediate its immunomodulatory effects (Clark et al., 1996, 1997).

CA125 (MUC 16) is the largest mucinous glycoprotein in the human genome, coding for ~24,000 amino acids (Yin et al., 2002). It is best known for its role as a specific marker for epithelial ovarian cancer (Bast et al., 1981, 1983). CA125 isolated from the human ovarian cancer cell line, OVCAR-3, is heavily N- and O-glycosylated, and its constituent glycans have been sequenced (Kui Wong et al., 2003). CA125 is secreted by endometrial epithelial cells during the same temporal window of the menstrual cycle as GdA (Kui Wong et al., 2003). Like GdA, this mucinous glycoprotein also becomes a major secreted product during the first trimester of human pregnancy. CA125 derived from OVCAR-3 cells suppresses cytotoxicity mediated by NK and lymphokine activated killer cells (LAK) *in vitro* when present at the physiological concentrations seen in the endometrium and decidua during the first trimester of pregnancy (Patankar et al., 2005). CA125 manifests this specific effect by blocking NK cell synapse formation, which results in the direct inhibition of NK cell mediated cytotoxicity (Gubbels et al., 2010).

In summary, GdA and CA125 likely participate in suppressing the maternal immune responses before implantation and continue to do so until mid-trimester. Defective expression of these glycoproteins during this stage of pregnancy would likely result in implantation failure or early pregnancy loss. However, whether defective expression of these modulators sets the stage for other pathological processes that are manifested after midtrimester is currently unknown.

### Differential expression of human major histocompatibility (MHC) antigens

The human leukocyte antigen (HLA) region of human chromosome 6 encodes many immune system genes, including the MHC complex class I and II molecules, which can be found on the surface of almost all nucleated cell types. HLA expression is tightly regulated at the feto-maternal interface, perhaps because direct engagement of foreign paternal or maternal antigens could trigger fetal rejection. When the human embryo first makes contact with the maternal endometrial epithelium, placental trophoblast cells at the adhesion site fuse and form a syncytium of multinucleated cells called syncytiotrophoblast (SynT) cells. Unlike normal cells and tissues, SynT and the underlying villous cytotrophoblasts (CytoT) do not express HLA class I and class II molecules (Hutter et al., 1996). This unusual characteristic ensures that paternal HLA antigens that could trigger histocompatibility based immune rejection are not expressed on these invasive trophoblast populations.

This lack of HLA expression, however, is somewhat problematic from an immunological perspective. About 70% of the immune cells at the implantation site during the early stages of pregnancy are uNK cells (King et al., 1996). Though not as potent in this activity as peripheral NK cells, uNK cells lyse HLA negative cells based on the “missing self” hypothesis (King et al., 1989; Karre, 1991). Currently, there is no explanation for how SynT resist these NK cell-mediated responses, since they are somewhat sensitive to lymphokine activated killing (King and Loke, 1990). However, there are large amounts of GdA and CA125 in the uterus during implantation that could substantially affect uNK cell mediated cytotoxicity.

**Figure 2:**
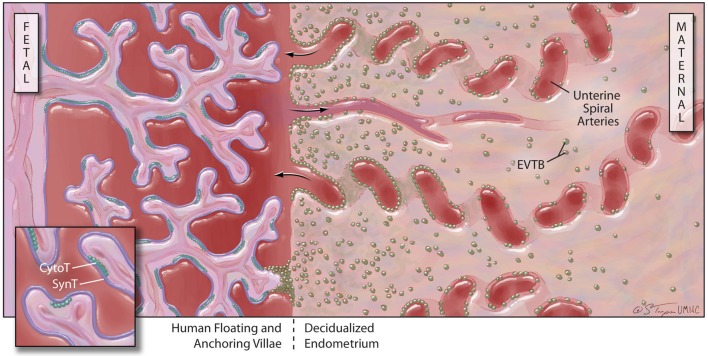
**Human placental structure (after 12 weeks of gestation): the human placenta has a fetal and a maternal side.** The fetal side consists of a mass of tree-like villous structures that are bathed in maternal blood. Unlike floating villae, anchoring villae traverse the blood-filled intervillous space and attach to the maternal decidualized endometrium. The maternal decidua is populated by stromal and immune cells and is crossed by spiral arteries that dump blood into the intervillous space. Floating and anchoring placental villae are coated by an inner layer of individual, fetally-derived cytotrophoblast (Cyto-T) cells and an outer layer of fused syncytiotrophoblast (Syn-T) cells. A third population of fetally-derived trophoblast cells arises from Cyto-T at the tips of anchoring villae. These extravilous cytotrophoblast (EVTB) cells invade deeply into the maternal tissues and remodel maternal spiral arteries.

Further complexity in this system is added by the expression of uncommon HLA class I molecules on specific, highly-invasive trophoblast cell subpopulations. The placental villi are a complex series of branching structures that contain a core of fetal vessels surrounded by stroma (Figure 2). Separating the stroma and fetal vessels from the maternal blood present in the spaces between villous structures are an inner, non-continuous layer of villous cytotrophoblast cells (CytoT) and an outer, continuous layer of fused, multinuclear SynT cells (Georgiades et al., 2002). Some placental villae float freely in the intervillous space, while others traverse the space to attach or anchor themselves to the maternal decidualized endometrium (decidua). The third trophoblast cell type, extravillous cytotrophoblast (EVTB) cells arise at the tips of anchoring villae and invade deeply into the maternal decidua (Benirschke, 1994). Invasion in humans typically extends into the inner third of the uterine muscle (myometrium). EVTB also invade the maternal uterine vessels, called spiral arteries due to their anatomic appearance. EVTB plug maternal spiral arteries until approximately 10 weeks of pregnancy (Burton and Jauniaux, 2004) and remodel the vessel walls to convert them from highly vasoactive structures into relatively unresponsive conduits for blood flow from mother to baby. After 10 weeks of gestation, EVTB vascular plugs disappear to allow unobstructed flow through these conduits (Meekins et al., 1997).

EVTB are unlike all other human cells in their MHC class I expression. HLA-G and HLA-E are MHC class Ib molecules with restricted polymorphism that can be detected on the surface of EVTB (Juch et al., 2012). uNK cells bear inhibitory killer immunoglobulin-like receptors (KIR) that specifically recognize these molecules. HLA-E binds to CD94/NKG2A (King et al., 2000), whereas HLA-G binds to KIR2DL4 (Rajagopalan and Long, 1999). EVTB also express HLA-C, which is a polymorphic MHC class I molecule like HLA-A and HLA-B and could—but does not appear to—strongly stimulate harmful antipaternal adaptive immune responses (Chazara et al., 2011). HLA-C molecules also specifically bind to NK cell KIRs.

### Other immunological activities of HLA-G

**Figure 3:**
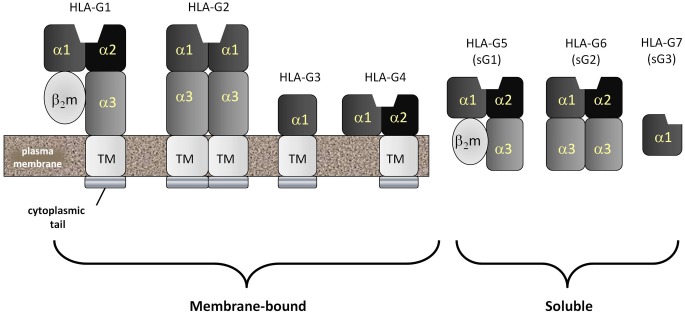
**HLA-G: the most common form of the non-classical MHC class Ib molecule, HLA-G, mimics HLA-A and -B in structure and is called HLA-G1.** HLA-A, -B and -G1 are all homodimers of an MHC class I heavy chain comprised of five domains and a stabilizing second molecule, beta-2 microglobulin (β_2_m). The MHC class I heavy chain consists of an α1 and α2 domain (forming the antigenic peptide-binding groove), an α3 domain, a transmembrane domain and a cytopalasmic tail. Unlike classical MHC class I molecules, the cytoplasmic tail of HLA-G is very short, containing only six amino acids. Also unlike classical MHC class Ia molecules, HLA-G can be detected as several spliced variants. The most common of these are the membrane-bound HLA-G1, -G2, -G3 and -G4 and the soluble HLA-G5, -G6 and -G7. Soluble forms have lost their transmembrane segments and cytoplasmic tails during splicing.

Currently, seven different isoforms of HLA-G, designated G1–G7, have been identified. HLA-G1, -G2, -G3 and -G4 are membrane-associated forms whereas HLA-G5, -G6 and -G7 (Figure 3) are soluble forms (Favier et al., 2007b). Both soluble and membrane associated forms of HLA-G can induce many immunomodulatory effects *in vitro* (Hunt, 2006; Favier et al., 2007b). These include: (1) inhibition of NK cell-mediated responses by either soluble or cell surface associated forms; (2) abrogation of the lytic activity mediated by CTLs via either soluble or membrane bound isoforms; (3) suppression of the IFN-γ mediated upregulation of CD8α mRNA by recombinant soluble forms of HLA-G1 and HLA-G2; (4) inhibition of the alloproliferative responses of CD4^+^ T cells as a membrane-bound molecule expressed by third party inert cells or by cells presenting a stimulating antigen; (5) induction of the differentiation of Tregs by stimulating antigen-producing cells; and (6) disruption of DC maturation. These effects are mediated through direct binding to inhibitory receptors designated immunoglobulin-like transcript-2 and -4 (ILT2, ILT4) and the KIR, KIR2DL4 (Favier et al., 2007a). ILT2 is expressed by both myeloid and lymphoid cells. ILT4 is exclusively expressed by myeloid cells and KIR2DL4 is expressed by NK and some T cells bearing CD81.

The effects of HLA-G *in vitro* are quite varied and affect many different types of potential immune responses in the pregnant uterus. However, the exact protein levels of the soluble and cell surface-associated isoforms of HLA-G in the fetoplacental unit and in the maternal decidua and periphery have yet to be determined. There also remain questions about HLA-G expression in different physiological states, including the proposal that the soluble forms of HLA-G (HLA-G5, -G6, and -G7) are not present in the pregnant uterus at all (Blaschitz et al., 2005; Sargent, 2005). In fact, the functions of HLA-G during the early human implantation have been difficult to definitively establish. Specifically, decidual NK (dNK) cells do not reproducibly express KIR2DL4 and the interaction between this KIR and HLA-G has not been consistently demonstrated (Apps et al., 2008). In addition, Moffet et al. have recently shown that HLA-G has no effect on freshly isolated human dNK cells in several functional tests that are known to greatly affect peripheral blood NK cells (Apps et al., 2011).

### Regulatory T cells (tregs)

Tregs are essential for the development of immune privilege in the uterus during early but not late mouse pregnancy (Aluvihare et al., 2004; Shima et al., 2010). In humans, these cells have a specific phenotype (CD4^+^CD25^+^FoxP3^+^) and accumulate in the endometrium during the follicular phase of the menstrual cycle (Arruvito et al., 2007). These numbers are maintained if implantation proceeds, but otherwise decrease dramatically in the luteal phase. A decrease in the number of decidual Tregs during early pregnancy in women is correlated with spontaneous abortions (Sasaki et al., 2004). Tregs decrease the cytolytic activity of NK cells (Ghiringhelli et al., 2006), impede the development of DCs (Bluestone and Tang, 2005) and negatively impact the proliferation of and cytokine release by CD3^+^ T cells (Earle et al., 2005).

### Galectins

Galectins are a family of small lectin molecules that generally have a universal affinity for N-acetyllactosamine (galactose in β1-4 linkage to N-acetylglucosamine; Galβ1-4GlcNAc) but which can, in some cases, bind to other carbohydrate sequences (Barondes et al., 1994). For example, several galectins, including galectin-3, also bind to the Thomsen–Friedenreich (T–F) antigen (Galβ1-3GalNAc) (Bian et al., 2011). In 1983, placental protein 13 was the first galectin isolated from the female reproductive tract (Bohn et al., 1983). Galectin-1 is another major galectin that is found in villous CytoT. This galectin has been reported to induce T cell apoptosis (Perillo et al., 1995), although there is some debate about whether this effect was due to non-physiological incubation conditions (Stowell et al., 2007). Nonetheless, interest in the expression of human placental galectins increased substantially when galectin-1 deficient mice in a stress-induced model of pregnancy failure were reported to display higher rates of fetal loss than control mice (Blois et al., 2007). These investigators also demonstrated that this effect could be completely reversed by the administration of recombinant galectin-1. Galectin-1 has been proposed to mediate its effects via a variety of different mechanisms including: (1) differentially regulating the survival of Th cell subsets (Toscano et al., 2007; Motran et al., 2008); (2) controlling T cell trafficking (He and Baum, 2006; Norling et al., 2008); and (3) promoting the differentiation of tolerogenic DCs (Ilarregui et al., 2009; Kuo et al., 2011). In one recent study, compelling evidence was provided that suggests the overall serum level of galectin-1 is decreased dramatically (eight-fold) in spontaneous abortion patients when compared to normal pregnant controls (Tirado-Gonzalez et al., 2013).

### Indoleamine 2,3-dioxygenase (IDO)

Indoleamine 2,3-dioxygenase (IDO) is an enzyme that can be induced in specific macrophages following their stimulation with IFN-γ and other mediators (Munn and Armstrong, 1993). Activation of this enzyme inhibits T cell-mediated responses by catabolizing the tryptophan that is essential for normal T cell proliferation. IDO is also synthesized by human Syn-T isolated from fresh placenta (Kamimura et al., 1991). Based on these effects, this enzyme was proposed to be a major mediator of immune tolerance during pregnancy. To test this hypothesis, Munn et al. exposed pregnant mice carrying either syngeneic or allogeneic fetuses to 1-methyl-tryptophan, a pharmacologic agent that inhibits IDO activity (Munn et al., 1998). Pregnant mice treated with this agent accommodated syngeneic but not allogeneic pups, suggesting IDO mediated tolerance. However, matings of male and female IDO-deficient mice generate litters of normal size, suggesting that the expression of IDO is not absolutely obligatory for mouse reproduction (Baban et al., 2004). Nonetheless, IDO likely plays a complementary role to other pregnancy-specific factors that insure the suppression of responses against the semiallogeneic fetus.

### Uromodulin

Tamm–Horsfall glycoprotein (THP) is the major protein/glycoprotein component in human urine (Tamm and Horsfall, 1950). Uromodulin is a differentially glycosylated form of THP that is present in the urine of pregnant human females, but not human males or non-pregnant females (Easton et al., 2000). It is 13 times more potent in the inhibition of antigen-induced T cell proliferation *in vitro* than THP derived from non-pregnant females and males (Hession et al., 1987). Rigorous analysis of uromodulin-derived oligosaccharides revealed the presence of unusual core 2 type O-glycans terminated with up to three SLEX terminals (Easton et al., 2000). By comparison, THP from non-pregnant females and males expresses very simple mono- and di-sialylated derivatives of Core 1 O-glycans, indicating a dramatic remodeling of THP glycosylation. Analysis of THP O-glycans isolated from females 2 months postpartum revealed a near total loss of SLEX modifications and a reversion back to forms that characterize the non-pregnant state. This observation suggests that uromodulin expression relies on human pregnancy hormones (e.g., estrogens, progesterone) or other related factors. How uromodulin contributes to the maintenance of immune privilege during pregnancy remains an enigma.

## Immune privilege for semen and sperm in the female reproductive tract

The immune system in the human female reproductive tract is also challenged by both the cellular and soluble components of human semen. The vagina and the cervix represent a relatively hostile environment for human sperm (Drobnis and Overstreet, 1992). Soon after sexual intercourse, maternal neutrophils, monocytes, and lymphocytes are released from the cervical epithelium during a cellular response known as *the leukocyte reaction* (Pandya and Cohen, 1985; Thompson et al., 1992). Human sperm do not express paternal MHC class I or II molecules on their surface and should not trigger histocompatibility-based responses (Hutter and Dohr, 1998). However, immature germ cells, epithelial cells, and leukocytes are present in semen, although they usually comprise less than 15% of the total cellular fraction (Fedder, 1996). Unlike sperm, these cells express paternal MHC molecules that could stimulate an MHC-restricted response at the surface of the cervico-vaginal epithelium (Zinkernagel and Doherty, 1974).

Sperm arise from testicular germ cells after the commencement of puberty and long after the period of thymic education (Fijak and Meinhardt, 2006). Proteins that are unique to human sperm are therefore foreign to the immune system and could be classified as either autoantigens or neoantigens. Evidence favoring this categorization includes the known induction of autoimmune orchitis following the autologous injection of testicular homogenates at sites distal to the testes (Tung et al., 1981). However, allografts and xenografts resist rejection following transplantation into the testis itself (Head and Billingham, 1985). This immune privileged state was initially thought to be due to the presence of a blood-testis barrier that protected germ cells from immune effector cells and antibodies (Setchell, 1967; Dym, 1973). Arguing against this paradigm is the fact that germ cell neoantigens on spermatogonia and early spermatocytes can be found in the basal compartments of the testis, which lack the blood-testis barrier (Yule et al., 1988; Saari et al., 1996). Further, neoantigens can also be detected in seminal plasma, which contains components that are produced by several glands in the male urogenital tract (Gupta et al., 1988).

Many of the known sperm and seminal plasma neoantigens are produced in response to androgen stimulation. While these antigens should provoke a potent immune response in the female reproductive system, the incidence of women with antisperm antibodies is only 2–3%. When antibodies are produced, however, subfertility or infertility often follows (Rumke and Hellinga, 1959; Lombardo et al., 2001). Allergic reactions to the fluid components of seminal plasma are also rare (Sublett and Bernstein, 2011). These results suggest that powerful immune-deviating effects are in play within the female genital tract.

Based on the excellent outcomes of IVF and artificial insemination procedures, which separate germ cells from seminal plasma, it is apparent that seminal plasma components are not required for successful fertilization. On the other hand, exposure to seminal plasma may be crucial to reductions in certain disease states in humans. Preeclampsia is a common but incompletely understood complication of pregnancy with pleomorphic pathological effects (Pennington et al., 2012). Interestingly, women who have had prolonged exposure to semen via unprotected oral or vaginal sex exhibit a considerably lower risk of developing preeclampsia than women who have had a much more limited duration of semen exposure (Basso et al., 2001; Kho et al., 2009). These results lead to the conclusion that seminal plasma suppresses specific immune responses in the female reproductive tract and, in several instances, tolerizes the female to paternal immune challenges. The net effect would be to protect the female from other immune-related disease states of pregnancy. Several factors in seminal plasma have been proposed to be responsible for this immune deviation.

### Prostaglandins

Human seminal plasma contains very high concentrations of prostaglandins when compared to other bodily secretions. These bioactive compounds were initially independently identified in this fluid by von Euler and Goldblatt in 1935 (Goldblatt, 1935; von Euler, 1935, 1936). Prostaglandin E_1_, E_2_, E_3_, F_1α_, and F_2α_ have been detected in human seminal plasma (Samuelsson, 1963). It is now apparent that PGE_2_, 19-hydroxyprostaglandin E_1_ and 19-hydroxyprostaglandin E_2_ are the three major prostaglandins in human seminal plasma, each being present in millimolar concentrations (Taylor and Kelly, 1974; Kelly et al., 1976; Templeton et al., 1978). Since these lipid mediators often manifest their effects in the μM to nM concentration range, virtually all pathways that are affected by these lipids are operating under saturating conditions in human semen.

PGE_2_ is a potent modulator of immune function. The effects of PGE_2_ have been the subject of intense investigation for over 20 years because of its association with cancer and other pathological states. This prostaglandin can simultaneously manifest both proinflammatory and immunosuppressive effects. These effects are summarized with references in Table 2.

**Table 2:** **Effects of PGE_2_ on immune function**.

**Immunological effects**	**References**
Inhibits granulocyte functions	Smith, 1977
Limits the phagocytic activity of alveolar macrophages and their pathogen killing function	Hubbard et al., 2010
Promotes the tissue influx of neutrophils, macrophages, and mast cells	Yu and Chadee, 1998; Nakayama et al., 2006; Weller et al., 2007
Converts DCs to myeloid derived suppressor cells	Obermajer et al., 2011
Suppresses NK cell mediated cytotoxicity	Bankhurst, 1982; Goto et al., 1983
Inhibits NK cell responses to IL-12, IL-15, and IL-2	Joshi et al., 2001; Walker and Rotondo, 2004
Blocks NK cell production of IFN-γ, inhibiting NK cell helper function	Mailliard et al., 2005
Disrupts early stages of differentiation of dendritic cells (DCs)	Kaliński et al., 1997
Promotes the induction of mast cells and their local attraction and degranulation	Hu et al., 1995; Gomi et al., 2000
Directly inhibits T cell production of IL-2 and IL-2 responsiveness	Walker et al., 1983
Enhances the production of Th2-attracting chemokines	McIlroy et al., 2006
Supports the induction of fully mature DCs	Jonuleit et al., 1997
Accelerates DC maturation and elevates their costimululatory molecules when present in combination with IL-1β and TNF-α	Rieser et al., 1997; Kaliński et al., 1998
Promotes the expression of CCR7, the receptor for chemokines L19 and L20 in monocyte-derived DCs	Luft et al., 2002; Scandella et al., 2002
Inhibits early stages of B cell activation and Ig class switching	Simkin et al., 1987
Limits migration of DCs via induction of tissue inhibitor of proteinase-1	Baratelli et al., 2004
Increases the expression of IL-10, thrombospondin and IDO in DCs	Kaliński et al., 1997; Doyen et al., 2003; Braun et al., 2005
Promotes the maturation of DCs with an impaired ability to induce CTL-, Th1- and NK cell-mediated type 1 immunity	Kaliński et al., 1998, 1999; Gustafsson et al., 2008
Suppresses the level of bioactive IL-12p70	Kaliński et al., 1998
Blocks the ability of DCs to attract naïve T cells	Muthuswamy et al., 2010
Suppresses the production of IL-12 in monocytes and DCs	van der Pouw Kraan et al., 1995; Kaliński et al., 1997, 1998
Blocks the expression of the IL-12 receptor in monocytes and DCs	Wu et al., 1998
Promotes the development of IL-17 producing T cells	Sheibanie et al., 2007; Woolard et al., 2008; Boniface et al., 2009; Esaki et al., 2010
Inhibits cytotoxic T lymphocyte (CTL) activity	Lala et al., 1988; Parhar and Lala, 1988; Specht et al., 2001
Blocks activation of CTL responses by DCs by inhibiting IL-12 secretion	Watchmaker et al., 2010
Promotes IgE production	Carini et al., 1981
Promotes the development of regulatory T cells	Baratelli et al., 2005; Bergmann et al., 2007
Promotes the interaction of DCs with regulatory T cells	Muthuswamy et al., 2008
Required for the development of tumor associated suppressive macrophages and myeloid-derived suppressor cells	Heusinkveld et al., 2011; Obermajer et al., 2011
Induces the expression of IL-10 in tissue macrophages	Huang et al., 1998; Stolina et al., 2000
Suppress the production of retinoic acid in gut-associated DCs	Stock et al., 2011

The overall effects of PGE_2_ include: (1) inhibition of responses mediated by phagocytic cells (neutrophils, macrophages); (2) suppression of NK, CTL, and T helper type 1 responses; (3) activation of DCs but limitation of their ability to attract naïve, memory, and effector T cells; and (4) stimulation of the production of regulatory T cells and myeloid-derived suppressor cells. In summary, the effects of PGE_2_ are consistent with a role in the inhibition of antigen-driven Th1 responses and in the promotion of Th2 responses. This overall response is essentially that which would be necessary to suppress responses directed against neoantigens while simultaneously maintaining the effectiveness of select beneficial immune responses.

### Cytokine expression

Human seminal plasma contains substantial amounts of a potent immunoregulatory cytokine known as transforming growth factor-β (TGF-β) (Nocera and Chu, 1993), although only ~7% of TGF-β in seminal plasma is in the active form (Nocera and Chu, 1995). Latent TGF-β can be activated by acidic conditions (transient acidification to pH 3.2) (Wakefield et al., 1987). Still, even though the healthy vaginal environment is acidic at baseline, it is unlikely that a significant amount of TGF-β is activated here after deposition in the vagina because the buffering capacity of the relatively large volume of basic human seminal plasma causes the pH of the vaginal environment to increase from 4.3 to 7.2 within 8 s after ejaculation (Fox et al., 1973).

The levels of other cytokines in human seminal plasma have also been studied (Maegawa et al., 2002). The levels of IL-1α, IL-2, IL-4, IL-6, IL-8, TNF-α, interferon-γ, granulocyte colony-stimulating factor [G-CSF] and macrophage CFS [M-CSF] have been analyzed. The pro-inflammatory cytokines (IL-1α, TNF-α), chemokine (IL-8) and G-CSF are present, but at low levels. The remainder are undetectable. In short, the levels of inflammatory cytokines in the lower female genital tract are very low under normal physiological conditions.

Very elegant, but difficult to perform investigations have been conducted to assess the effects of seminal plasma on cytokine expression in the human cervix. Twelve hours after unprotected vaginal intercourse with ejaculation, the mRNA levels for colony stimulating factor 2, IL-6. IL-8 and IL-1α in human cervical biopsies are enhanced when compared to controls (abstention or condom-protected controls) (Sharkey et al., 2012b). Seminal fluid not only induces the expression of pro-inflammatory cytokines and chemokines in the cervix, but also causes a major influx of macrophages, DCs, and memory T cells (Sharkey et al., 2012b). Still, TGF-β has been the component of seminal plasma most directly implicated in this response (Sharkey et al., 2012a).

### Unusual glycosylation of human sperm and seminal plasma glycoproteins

The role of glycosylation in inducing immune privilege, particularly in the reproductive tract, has been understudied. Historically, carbohydrate ligands and their complementary lectin-like immune receptors have been difficult to isolate and characterize. However, the development of ultrasensitive mass spectrometric (MS) techniques for sequencing oligosaccharides, when combined with the use of glycan arrays to define carbohydrate binding specificities, have recently changed the research landscape in this area (Blixt et al., 2004; North et al., 2009). The molecular bases underlying the ability of specific carbohydrate sequences to act as functional groups that suppress immune function are now being revealed and previous predictions about these relationships are being validated (Clark et al., 1996, 1997).

Ultrasensitive MS profiling of the N-glycans associated with human sperm and seminal plasma has uncovered the expression of unusual glycans (Pang et al., 2007, 2009). A distinguishing feature of these glycans is the presence of Lewis^x^ and Lewis^y^ sequences that are rarely found on the oligosaccharides present on the surface of other normal cell and tissue types outside of the male reproductive system. These sequences are displayed in multivalent presentations on the terminal ends of biantennary, triantennary, and tetraantennary N-glycans (Figure 1). The N-glycans linked to the human ZP are similar, except that they are terminated with multivalent SLEX rather than Lewis^x^ or Lewis^y^ sequences (Pang et al., 2011).

The endogenous glycoprotein ligands for immune type lectins have been proposed to be the true mediators of immune homeostasis (Garcia-Vallejo and van Kooyk, 2009). There are four major glycoproteins in human seminal plasma that have been identified as endogenous ligands for DC-SIGN, an immune lectin associated with DCs. They include clusterin, galectin-3 binding protein, prostatic acid phosphatase, and protein C inhibitor (Clark et al., 2012). The exact function of these glycoproteins remains to be determined, but studies with parasites and other persistent pathogens that express Lewis^x^ and Lewis^y^ sequences suggest that they are likely involved in the induction of tolerance to the developing human *in utero* (Garcia-Vallejo and van Kooyk, 2009).

### Other factors in seminal plasma

A number of other seminal plasma factors display immunosuppressive effects *in vitro*. The primary assay that has been employed to assess this effect is the inhibition of phytohemagglutinin (PHA)-induced proliferation of T lymphocytes. Prostasomes are a group of 40–500 nm membranous vesicles secreted by the prostate into human semen. Prostasomes inhibit PHA-induced proliferation by 69% in a dose dependent manner (Kelly et al., 1991). However, human prostasomes contain galelectin-3, which could compete with phytohemagglutinin for binding to galactose-terminated glycans on T cell glycoproteins (Jones et al., 2010), so it is possible that this effect could involve a simple lectin blockade rather than a specific immunosuppressive effect. Whether prostasomes mediate a specific immunosuppressive effect remains to be verified.

Still another study indicated that human seminal plasma components with a MW >3.5 kDa also inhibit PHA-induced T lymphocyte proliferation (Ochsenkuhn et al., 2006). While seminal plasma glycoproteins could also inhibit PHA binding to T lymphocytes via non-specific lectin blockade, an antibody directed against TGF-β has been shown to inhibit this immunosuppressive activity by 50%, indicating that this specific cytokine could be partially responsible for this effect (Ochsenkuhn et al., 2006). The immune deviating effects of seminal plasma glycoproteins certainly deserve further attention.

Polyamines in seminal plasma have also been implicated in the suppression of immune responses in the female reproductive tract (Allen and Roberts, 1986). Spermine is present in millimolar concentrations in human seminal plasma (Agostinelli et al., 2007). Supplementation of lymphocyte cultures with spermine leads to a cytotoxic effect that mimics that seen after addition of seminal plasma (Allen and Roberts, 1987). Spermine inhibits LAK cell activity directed against cervical carcinoma cells by up to 60% at concentrations exceeding 10 nM (Evans et al., 1995). Although the precise mechanisms underlying this finding remain unclear, the deamination of spermine by amine oxidases generates both hydrogen peroxides and aldehydes that promote apoptosis (Agostinelli et al., 2007).

## Conclusions

Humans have a complex immune system consisting of both innate and adaptive arms and immune cells have developed intricate means of recognizing each other that involve HLA class I and class II molecules. Further complexity is introduced by the diversification of these molecules into many haplotypes to enable exceptionally precise recognition of self in the immunological context. However, this diversity may come at a cost, as it makes the paternal antigen-expressing human fetus the equivalent of a foreign organ transplant within the immunocompetent gravid female (Reisner et al., 2011). The maternal immune system cannot simply be inactivated to allow for reproduction because of the incumbent risk of infection, particularly that arising in the complex microbiologic milieu of the lower genital tract. A compromise state must therefore be established that will allow selective immune privilege for gametes and the developing fetus within the context of an otherwise immunocompetent female reproductive system. A reasonable, though not fully potent, immune response to pathogens must persist to protect the mother from infection.

The pathways that promote immune privilege are best understood in the eye (Streilein, 2003; Niederkorn, 2012). Niederkorn recently proposed an attractive hypothesis that suggests that metastatic uveal melanoma cells found in the liver have “plagiarized” the blueprints employed for ocular immune privilege to create “*ad hoc*” immune privileged regions in this distant site (Niederkorn, 2012). Obviously, there are enormous advantages for metastatic cells if this hypothesis is correct, as it likely is.

The proposal was made some time ago that a similar type of immune privilege exists for human gametes and the uteroplacental unit (Clark et al., 1996, 1997). Both SLEX and the Lewis^y^ carbohydrate sequences were originally defined as tumor-associated carbohydrate antigens, based on their specific expression on cancer cells but not on their progenitor cells (Abe et al., 1983; Fukushima et al., 1984). A recent study has confirmed the profligate expression of SLEX sequences on the human ZP (Pang et al., 2011). Lewis^y^ sequences are similarly expressed on human sperm and seminal plasma glycoproteins (Pang et al., 2007, 2009). The strong possibility exists that pathogens can also hijack those elements of immune privilege utilized in the reproductive system of humans to evade the immune response. For example, variants of *H. pylori* that express Lewis^x^/Lewis^y^ sequences on their lipopolysaccharides promote tolerance, whereas non-expressors evoke the severe inflammatory responses that result in the pathological symptoms associated with this bacterial pathogen (Bergman et al., 2006). HIV infection substantially increases the percentage of CD4^+^ and CD8^+^ T cells that express the Lewis^y^ sequence (Adachi et al., 1988; Kashiwagi et al., 1994), a characteristic that may allow for relatively unfettered viral proliferation.

It is a substantial challenge to understand how carbohydrate sequences act as functional groups to mediate immunomodulatory effects at the fetomaternal interface. One major obstacle has been the inability to sequence glycans from small amounts of glycoproteins, such as MHC class I molecules. Major advances in mass spectrometry have recently led to the complete structural analysis of native human ZP glycans by Dell and coworkers, a feat performed with only 5 μg of purified ZP (Pang et al., 2011). HLA-G expressed on EVTB is differentially glycosylated when compared to classical MHC molecules (McMaster et al., 1998). If HLA-G glycans act as crucial functional groups, then aberrant glycosylation could result in the pathological effects observed during pregnancy. Current molecular and proteomic strategies could never detect these glycobiological changes.

How the information in such carbohydrate signals is transmitted in immune cells must also be defined. Siglecs usually bear specific ITIM that mediate immune modulatory effects via conventional signaling mechanisms (Crocker et al., 2007). The two exceptions (Siglec H and Siglec 14) activate immune cells by interacting with the membrane protein, DAP12 (Angata et al., 2006; Blasius et al., 2006). Other C-type lectin-like receptor complexes are also expressed on NK cells, including the CD94/NKG2 family receptors that employ ITIM/ITAM (immunoreceptor tyrosine-based motif) dependent pathways (Borrego et al., 2006). Carbohydrate ligands for CD94 and NKG2 have not been defined, but neither have the glycans associated with naturally occurring MHC class I molecules that could interact with these lectins. The effects of galectins are more subtle and complex, because they can form lattices of different glycoproteins that promote signaling via both lectin-like and protein-protein interactions (Lau and Dennis, 2008). Since there are many galectins, the potential for establishing functional pathways by organizing such glycoprotein complexes on cell surfaces is substantial (Than et al., 2012).

In summary, these findings suggest that investigation of the pathways that evoke immune privileged states in humans could lead to an understanding of the mechanisms that enable pathogens and tumor cells to evade the immune response. Once such pathways are defined, they can hopefully be readily targeted for therapeutic intervention. The role of carbohydrate recognition in such processes is now beginning to be fully appreciated.

### Conflict of interest statement

The authors declare that the research was conducted in the absence of any commercial or financial relationships that could be construed as a potential conflict of interest.
